# A radiomic biomarker for prognosis of resected colorectal cancer liver metastases generalizes across MRI contrast agents

**DOI:** 10.3389/fonc.2023.898854

**Published:** 2023-02-02

**Authors:** Jianan Chen, Helen M. C. Cheung, Paul J. Karanicolas, Natalie G. Coburn, Guillaume Martel, Albert Lee, Chirag Patel, Laurent Milot, Anne L. Martel

**Affiliations:** ^1^ Department of Medical Biophysics, University of Toronto, Toronto, ON, Canada; ^2^ Sunnybrook Health Sciences Center, Toronto, ON, Canada; ^3^ Department of Medical Imaging, University of Toronto, Toronto, ON, Canada; ^4^ Department of Surgery, University of Toronto, Toronto, ON, Canada; ^5^ Department of Surgery, University of Ottawa, Ottawa, ON, Canada; ^6^ Division of General Surgery, The Ottawa Hospital, Ottawa, ON, Canada

**Keywords:** colorectal cancer, liver, metastasis, MRI, radiomics, contrast agents

## Abstract

**Introduction:**

Contrast-enhanced MRI is routinely performed as part of preoperative work-up for patients with Colorectal Cancer Liver Metastases (CRLM). Radiomic biomarkers depicting the characteristics of CRLMs in MRI have been associated with overall survival (OS) of patients, but the reproducibility and clinical applicability of these biomarkers are limited due to the variations in MRI protocols between hospitals.

**Methods:**

In this work, we propose a generalizable radiomic model for predicting OS of CRLM patients who received preoperative chemotherapy and delayed-phase contrast enhanced (DPCE) MRIs prior to hepatic resection. This retrospective two-center study included three DPCE MRI cohorts (n=221) collected between January 2006 and December 2012. A 10-minute delayed Gd-DO3A-butrol enhanced MRI discovery cohort was used to select features based on robustness across contrast agents, correlation with OS and pairwise Pearson correlation, and to train a logistic regression model that predicts 3-year OS.

**Results:**

The model was evaluated on a 10-minute delayed Gd-DO3A-butrol enhanced MRI validation cohort (n=121), a 20-minute delayed Gd-EOB-DTPA (n=72) cohort from the same institute, and a 5-minute delayed Gd-DTPA cohort (n=28) from an independent institute. Two features were selected: minor axis length and dependence variance. The radiomic signature model stratified high-risk and low-risk CRLM groups in the Gd-DO3Abutrol (HR = 6.29, p = .007), Gd-EOB-DTPA (HR = 3.54, p = .003) and Gd-DTPA (HR = 3.16, p = .04) validation cohorts.

**Discussion:**

While most existing MRI findings focus on a specific contrast agent, our study shows the potential of MRI features to be generalizable across main-stream contrast agents at delayed phase.

## 1 Introduction

Colorectal cancer is the 2nd leading cause of cancer deaths in North America (1). Many patients develop metastatic disease, with the liver being the most common site for metastases. In patients with colorectal liver metastases (CRLM), hepatic resection may potentially be curative (2). Contrast-enhanced MRI is routinely performed as part of preoperative work-up for patients with CRLM due to its high sensitivity and specificity (3, 4).

Gadolinium-based contrast agents (GBCA), including extracellular contrast agents (ECA) and hepatobiliary-specific contrast agents (HCA) have been widely used for liver imaging. ECA such as Gadopentetate dimeglumine (Gd-DTPA, Magnevist®) and macrocyclic gadobutrol (Gd-DO3A-butrol, GadovistTM (EU), Gadavist® (USA)) have been extensively utilized in the past two decades for liver MRI (5, 6). HCA on the other hand, for example Gadoxetic acid agents (Gd-EOB-DTPA, Primovist® (EU), and Eovist® (USA)), have been playing an increasingly important role in imaging CRLM because of their higher sensitivity in detecting liver lesions. As an HCA, Gd-EOB-DTPA demonstrates active uptake of contrast by hepatocytes leading to approximately 50% hepatobiliary excretion and 50% renal excretion, assuming a normal functioning liver (7). This active uptake leads to increased enhancement in hepatocytes; however, similar to ECAs, there remains a proportion of contrast that diffuses into the extracellular space on delayed phase (8).

The ability to accurately and non-invasively risk-stratify CRLM patients based on tumor characteristics may have important implications for personalized therapy, including treatment decision-making. Imaging biomarkers are attractive as they are non-invasive and can be readily implemented in clinical workflows as part of preoperative assessment. Radiomic biomarkers have been developed to predict CRLM prognosis from delayed-phase contrast enhanced (DPCE) MRIs. Late gadolinium enhancement of CRLM with both ECA and HCA have been shown to correlate with tumor fibrosis and overall survival in patients who had hepatic resection (9, 10). Radiomic features depicting the characteristics of CRLMs and liver parenchyma have been associated with pathological covariates and OS (11). However, the reproducibility and clinical applicability of these biomarkers are limited due to the variations in MRI protocols between hospitals and the lack of independent validation in external datasets (12). Factors including choice of contrast agent, timing of delayed image acquisition, and scanner types could have considerable impacts on enhancement patterns of CRLMs but they are rarely taken into account when developing biomarkers (13).

The purpose of this study is to identify radiomic features of DPCE MRI that are relatively robust across contrast agents, and investigate whether a radiomic signature built based on these features, when developed on a single contrast-agent, is generalizable to other types of contrast agents for the prediction of OS in CRLM patients.

## 2 Materials and methods

**Figure 1:**
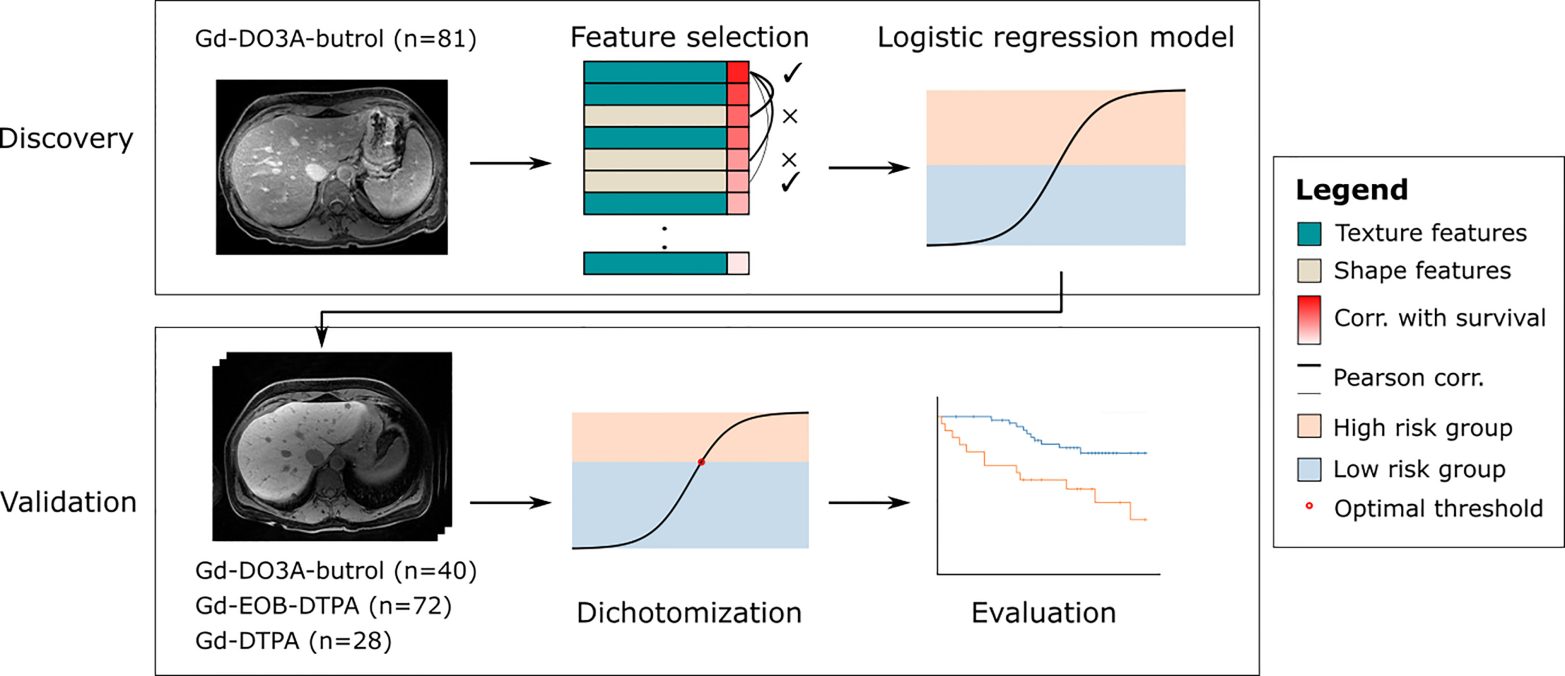
Overview of the data split and the general discovery and validation workflow.

This is a retrospective study performed on three cohorts of CRLM patients, including two previously described Gd-DO3A-butrol-enhanced and Gd-EOB-DTPA-enhanced MR imaging cohorts from the same institute and a new Gd-DTPA-enhanced MRI cohort from an independent institute. The Gd-EO3A-butrol cohort was randomly split (stratified by OS events) into a discovery cohort (n=81) and a validation cohort (n=40). The Gd-EOB-DTPA (n=72) and Gd-DTPA cohorts (n=28) were used for evaluation only (**Figure 1**). 121 out of 130 Gd-EO3A-butrol patients, and 65 out of 72 Gd-EOB-DTPA patients have been previously reported (9, 10). The prior articles investigated the associations of tumor enhancement patterns with patient survival within individual contrast agents while in this manuscript we incorporate multiple cohorts from multiple institutes and use radiomics for contrast-agnostic biomarker discovery.

The review board at each institute approved the study and waived the requirement for written informed consent because the study design was retrospective and personal health information was deidentified.

### 2.1 Participants

**Figure 2:**
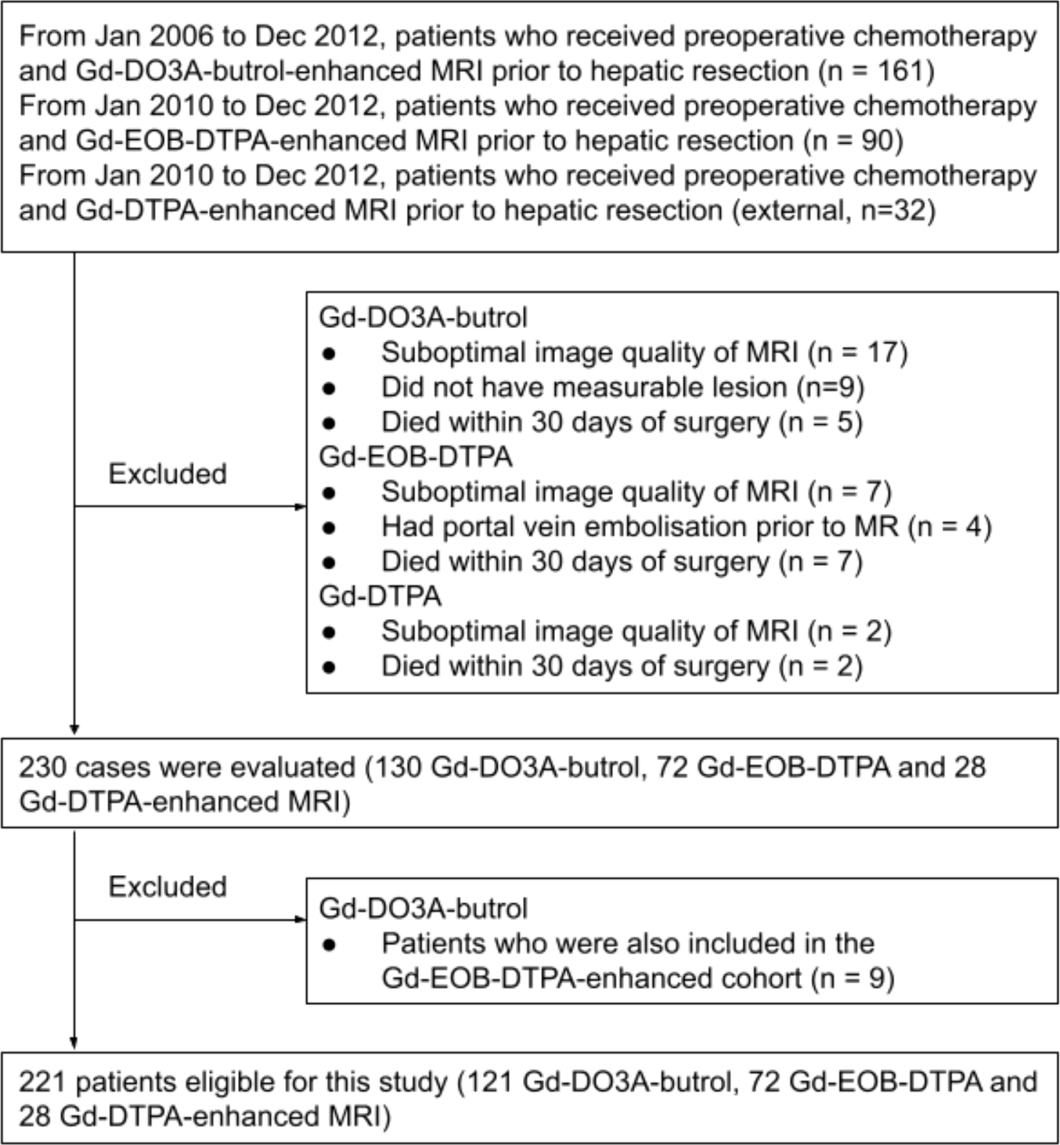
Flow chart of the inclusion and exclusion criteria.

Three retrospective patient cohorts were used in this study: patients who received preoperative chemotherapy (variable regiments determined by standard of care) and MRI with Gd-DO3A-butrol, Gd-EOB-DTPA, or Gd-DTPA enhancement prior to hepatic resection for colorectal cancer liver metastases between January 1, 2006 and December 31, 2012, between January 1, 2010 and December 31, 2012, and between January 1, 2010 and December 31, 2012, respectively (**Figure 2**)

In all three cohorts, patients were excluded for poor image quality, unmeasurable lesions according to Response Evaluation Criteria in Solid Tumors 1.1 (14), or surgery-related mortality. If multiple pre-surgical MRIs were available, the one closest to the surgical date was used. Patients with both Gd-DO3A-butrol and Gd-EOB-DTPA enhanced MRIs from the same institute (n=9) were assigned to the Gd-EOB-DTPA cohort.

Demographic information was obtained using the patient’s electronic patient record and publicly available obituary databases. The following clinical information was obtained: age, sex, chemotherapy prior to MRI, number of tumors, time from diagnosis of primary to diagnosis of metastasis, node positivity of primary colorectal tumor, carcinoembryonic antigen (CEA) level, and overall survival. The primary endpoint for this study was overall survival (OS).

### 2.2 MRI examination

Gd-DO3A-butrol and Gd-EOB-DTPA enhanced MRIs were acquired using standard institutional clinical liver imaging protocols. Delayed 3D axial T1 imaging was performed with 10-min post-intravenous injection of Gd-DO3A-butrol (0.1ml/kg body mass up to 10ml at 1.0mmol/ml) and 20-min post-intravenous injection of Gd-EOB-DTPA (10ml of 0.25mmol/ml). Scans were performed on 1.5-T (GE Twinspeed™, TR, 4.5; TE, 2.2; flip angle, 15; slice thickness, 5mm; spacing, 2.5mm; FOV, 380mm; matrix, 320×192) or 3.0-T (Philips Achieva™, TR, 3.0; TE, 1.4; flip angle, 10; slice thickness, 3mm; spacing, 1.5mm, FOV, 380; matrix, 250×250) magnets with an eight-channel body phased array coil covering the entire liver. Further details are given elsewhere (9, 10). Gd-DTPA enhanced MRI were acquired using delayed 3D axial T1 imaging at 5-min post-intravenous injection of Gd-DTPA (10-20mL of 0.5mmol/mL), on 1.5-T (Siemens SymphonyTim™, TR, 4.3; TE, 1.4; flip angle, 18; slice thickness, 2.5mm; spacing, 1.25mm; matrix, 320×132) or 1.5-T (Siemens TrioTim™, TR, 3.5; TE, 1.3; flip angle, 11; slice thickness, 2mm; spacing, 1.125mm, matrix, 320x144) magnets with a phased array coil covering the liver.

### 2.3 MRI lesion segmentation

For cohorts with Gd-DO3A-butrol and Gd-EOB-DTPA enhanced MRI, segmentations were performed on ClearCanvas, an open source DICOM viewer (http://clearcanvas.github.io/), by HC (with 7 years of experience). The images and segmentation files were converted into the NIfTI file format for further analysis. For the cohort with Gd-DTPA-enhanced MRI, image segmentations were performed in ITK-Snap v3.6.0 (15), by AL (with 1 year of experience). Segmentations were performed on the 10-minute delayed phase sequence in the Gd-DO3A-butrol cohort, 20-minute delayed phase sequence in the Gd-EOB-DTPA cohort, and 5-minute delayed phase sequence in the Gd-DTPA cohort. The readers were blinded to all clinical information except for history of CRLM at time of segmentation.

### 2.4 Image preprocessing and analysis

Radiomics features were extracted using the pyradiomcs (v3.0.1) package. The delayed 3D axial T1 images were interpolated using B-spline interpolation (SimpleITK v1.1.0) with the resampled pixel spacing of 1, 1, 1mm. The resampled image intensities were z-score normalized, scaled by 100 and discretized with the bin size of 5. The preprocessed image was inputted into pyradiomics (v3.0.1) for feature extraction. 100 features describing intensity, shape and texture were extracted. For patients with multiple metastases, features of the largest lesion were extracted.

### 2.5 Feature selection

**Figure 3:**
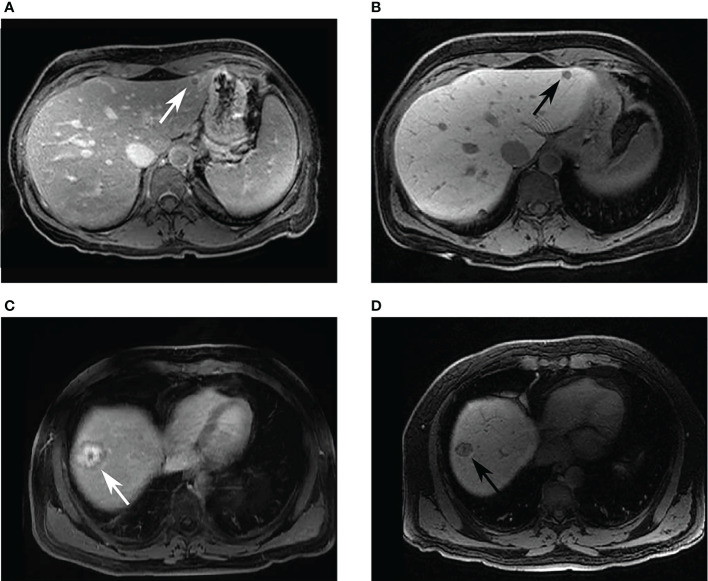
Example images of patients who had received both Gd-DO3A-butrol and Gd-EOB-DTPA-enhanced imaging. Two patients with colorectal cancer liver metastases. A 44-year-old female patient with a hypointense lesion in both **(A)** 10-minute delayed Gd-DO3A-butrol-enhanced T1 axial image, and **(B)** 20-minute delayed Gd-EOB-DTPA-enhanced T1 axial image. A 58-year-old male patient with 3-layer tumor enhancement pattern in both **(C)** 10-minute delayed Gd-DO3A-butrol-enhanced T1 axial image and **(D)** 20-minute delayed Gd-EOB-DTPA-enhanced T1 axial image taken after 3 months. The patient received chemotherapy during the interval. MA: minor axis length, DA: gray level dependence variance.

Scans from patients who received both Gd-EO3A-butrol and Gd-EOB-DTPA enhanced MRI were evaluated to select radiomic features that are contrast-agent-agnostic (**Figure 3**). We discovered that scans across contrast-agents vary little in size but vary greatly in intensity. We also observed that second and higher-order texture features are relatively stable across contrast agents compared to intensity features, as expected since texture features are calculated based on local texture changes rather than absolute intensity values. First-order intensity features were discarded as they were heavily influenced by contrast agents, while shape features and texture features were further analyzed.

A rank for each feature was calculated based on the average rank of the Mann-Whitney U-statistic in five stratified (by OS) cross-folds of the discovery dataset (Gd-DO3A-butrol, n=81), comparing feature distributions in patients with and without OS events. We then selected the feature with the highest overall ranking, and found the next best feature from the other feature category that is not correlated with the selected feature (Pearson correlation p>0.05), in order to reduce overfitting and maximize feature diversity. As a baseline comparison, we also performed least absolute shrinkage and selection operator (LASSO) feature selection across the Gd-DO3A-butrol discovery dataset (n=81) for association with OS events.

### 2.6 Statistical analysis

A radiomic signature was built using logistic regression to evaluate the predictive performance of the selected features. The model was trained on the discovery cohort and applied to the validation cohorts for evaluation. Model predictions were dichotomized at the default value of 0.5 in the Gd-DO3A-butrol validation cohort. To account for the shift in feature distributions with different contrast agents, which may lead to lower precision and recall if the same model cutoff is used, thresholds for the Gd-EOB-DTPA and Gd-DTPA cohorts were determined using the Maximally Selected Rank Statistics algorithm (16). Log-rank tests were used to test associations between patient OS and radiomic signature groups. Cox proportional hazards modeling was used to evaluate associations between OS and signature groups, alone and in combination with clinical covariates. The radiomic signature groups were compared to other radiomic biomarkers, including models trained with LASSO selected features, and a recent clinical-radiomic model for Gd-EOB-DTPA MRI (17).

All statistical analysis was implemented in R (v3.6.3), using base-R functions and package survival (v3.2-7).

## 3 Results

A total of 230 DPCE MRI scans from 221 CRLM patients (128 male, 93 female; mean age ± standard deviation, 63 ± 11 years; age range, 30-86 years) were included in this study (**Table 1**). 111 out of 221 patients (50%) had more than one lesion. Median follow-up was 40 months (range, 2-107 months). 69 deaths (out of 221, 31%) occurred during the follow-up period. 34 out of 179 patients with available clinical annotations (19%) had Fong clinical risk scores larger or equal to 3 (18).

**Table 1:** Demographics of the study population (n = 221).

	Gd-DO3A-butrol discovery cohort^†^	Gd-DO3A-butrol validation cohort^†^	Gd-EOB-DTPA cohort^‡^	Gd-DTPA cohort^‡ϕ^
Parameters	Value			
No. of patients	81	40	72	28
Male	45 (56)	25 (63)	38 (53)	20 (71)
Female	36 (44)	15 (37)	34 (47)	8 (29)
Age (y): mean ± SD	65 ± 11	64 ± 11	61 ± 13	62 ± 9
Clinical risk score				
<3	61 (84)*	30 (81)*	54 (75)*	–
≥3	12 (16)*	7 (19)*	15 (25)*	–
Not available	8 (10)	3 (8)	3 (4)	28 (100)
Number of tumors				
=1 tumor	48 (57)	21 (53)	27 (38)	14 (50)
>1 tumor	33 (43)	19 (47)	45 (62)	14 (50)
Size of largest tumor				
< 5cm	67 (83)	31 (78)	60 (83)	23 (75)
≥ 5cm	14 (17)	9 (22)	12 (17)	7 (25)
OS event	27 (33)	13 (33)	18 (25)	11 (39)

Unless otherwise specified, data are numbers of patients, with percentages in parentheses. OS, overall survival. Clinical risk score is the Fong risk score.

*Node positive status was missing for some patients, resulting in incomplete clinical risk score. The percentages for risk scores are therefore calculated based on patients who have complete clinical data.

^†^Used as a discovery set to identify features associated with survival and train models.

^‡^Used as independent validation sets.

^ϕ^External dataset.

### 3.1 Generalizable radiomic features selection

We first extracted 100 quantitative features describing tumor characteristics from each scan. Features describe tumor intensity (n=18), shape (n=22), and texture (n=68). We reason that since all intensity features (n=18) are sensitive to changes in the absolute value of voxel intensities, which is naturally influenced by contrast agent choice and other imaging parameters, they should be excluded upfront to prevent overfitting to these parameters and impeding generalizability.

We then performed feature selection on the remaining features to discover those that were associated with patient OS. In the discovery cohort of Gd-DO3A-butrol patients (n=81), we ranked features based on their average association with OS in 5 cross-folds. The best performing feature was minor axis length^1^ (shape feature; HR=1.50, p=.001, log-rank test). To diversify feature selection, we looked for the next best performing feature that describes tumor texture that is not correlated with minor axis length. We calculated the threshold of significant correlation as Pearson correlation larger or equal to 0.3, based on sample size of 81 at alpha of.05 and power of 0.8. As a result, the second selected radiomic feature was dependence variance^2^ (texture feature; HR=1.43, p=.01, log-rank test; correlation with minor axis, r=0.23, Pearson correlation).

### 3.2 Radiomic signature is independent predictor of OS

Using the two radiomic features selected, we trained a radiomic signature using logistic regression on the discovery Gd-DO3A-butrol dataset. The intercept of the model is -0.86 and the coefficients are 0.48 for minor axis length and 0.15 for dependence variance, respectively. We then evaluated our radiomic signature on never-seen data, including the validation Gd-DO3A-butrol dataset, the Gd-EOB-DTPA dataset, and the Gd-DTPA dataset. We further dichotomized patients in each cohort into low- and high-risk groups for survival analysis.

**Figure 4:**
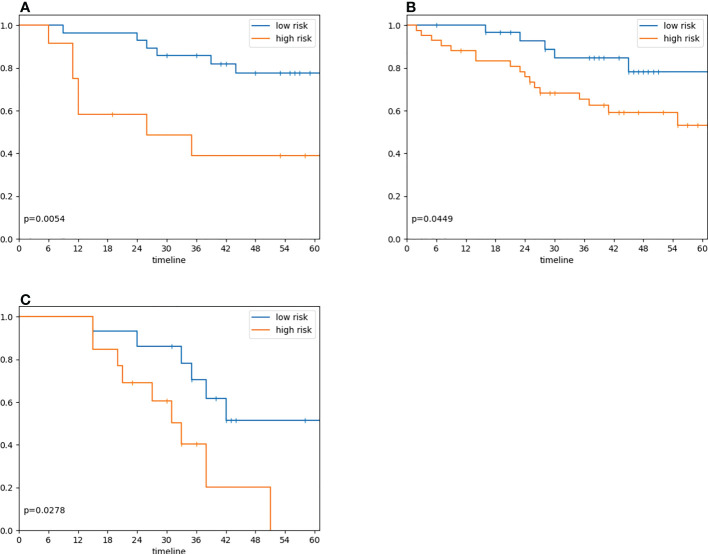
Radiomic signature risk group associations with overall survival. **(A)** Overall survival in 40 patients in the validation cohort who underwent preoperative Gd-DO3A-butrol-enhanced MRI, stratified by the radiomic signature model trained on Gd-EO3A-butrol training cohort (model score dichotomization threshold=0.5; log-rank test, p=.005). **(B)** Overall survival in 72 patients who underwent preoperative Gd-EOB-DTPA enhanced MRI, stratified by radiomic signature model (Maximally Selected Rank Statistic threshold=0.30; log-rank test, p=.04) **(C)** Overall survival in 28 patients who underwent preoperative Gd-DTPA enhanced MRI, stratified radiomic signature model (Maximally Selected Rank Statistic threshold=0.26; log-rank test, p=.03).

Our model successfully validated in the Gd-DO3A-butrol validation cohort. Patients in the high-risk group (n=18) had significantly lower 3-year survival rate than patients in the low-risk group (n=22) (**Figure 4A**; p=.005, log-rank test). Univariate analysis was performed to identify clinical covariates that are significant predictors of patient survival. The number of tumors was found to be significant in Gd-DO3A-butrol cohort (HR=4.11, 95% CI 1.19-14.14; p=.03; c-index=0.67), none of the covariates were significant in the Gd-EOB-DTPA cohort and age was significant in the Gd-DTPA cohort (HR=0.27, 95% CI, 0.09-0.79; p=.2, Wald test). We performed multivariable cox regression analysis with number of tumors, sex and our radiomic signature in all cohorts to test whether our radiomic signature provides added prognostic value of OS. In the validation cohort (**Table 2**), the combined model showed improved predictive power (c-index=0.72) over the clinical covariates alone and the radiomic signature remained independently predictive after adjusting for the clinical covariate (adjusted HR=13.54, 95% CI, 4.32-75.27; p=.003, Wald test).

**Table 2:** Cox regression model results for the association of radiomic biomarker with overall survival in the Gd-DO3A-butrol validation cohort.

Overall survival (n = 40)							
	Univariate analysis				Multivariable analysis (C-index = 0.72)		
Factor	c-index	HR	95% CI	P Value	HR	95% CI	P Value
Age (years)							
≥60	0.65	0.38	0.11, 1.29	.10	0.99	0.31, 3.15	.98
<60	–	–	–	–	–	–	–
Sex							
Male	0.54	0.40	0.09, 1.77	.23	–	–	–
Female	–	–	–	–	–	–	–
Number of tumors							
>1	0.57	1.57	0.57, 4.37	.38	7.03	3.38, 29.31	.007*
=1	–	–	–	–	–	–	–
Radiomic risk score							
High^†^	0.64	3.16	1.08, 9.19	.04*	13.54	4.32, 75.27	.003*
Low^†^	–	–	–	–	–	–	–

c-index, concordance index; HR, hazard ratio; CI, confidence interval.

*with p value that indicates statistical significance.

-denotes reference groups.

^†^high and low risk groups are determined using the default threshold for logistic regression models (0.5).

### 3.3 Validation in datasets with other contrast agents

We next evaluated our radiomic signature on cohorts that used different contrast agents to assess its generalizability. Despite being trained on the discovery Gd-DO3A-butrol dataset, our radiomic signature was associated with OS in both the Gd-EOB-DTPA cohort (**Figure 4B**; high-risk n=43, low-risk n=29, p=.04, log-rank test) and Gd-DTPA cohort (**Figure 4C**; high-risk n=11, low-risk n=17, p=.03, log-rank test). Similar to the Gd-DO3A-butrol validation cohort, in both the Gd-EOB-DTPA (**Table 3**) and the Gd-DTPA (**Table 4**) cohorts. In the Gad-EOB-DTPA cohort, none of the clinical variates were significant, and the radiomic signature is the only significant predictor of survival (HR=3.54, 95% CI, 1.53-8.21; p=.003, Wald test, c-index=0.66). Combining the radiomic signature with clinical covariates improved predictive power (c-index=0.69). In the Gad-DTPA cohort, combing radiomic signature (HR=3.16, 95% CI, 1.08-9.19, p=0.04, Wald test, c-index=0.64) with clinical covariates also improved predictive power (c-index=0.74). The radiomic signature remained a significant independent predictor of OS after accounting for clinical factors in both cohorts, with adjusted hazard ratio of 3.23 (95% CI, 1.38-7.65; p=.008, Wald test) in the Gd-EOB-DTPA cohort and adjusted hazard ratio of 7.78 (95% CI, 1.79-33.73; p=.006, Wald test) in the Gd-DTPA cohort. This demonstrates that radiomic signatures could generalize across contrast agents when features are selected considering robustness across contrast agents and feature diversity.

**Table 3:** Cox regression model results for the association of radiomics biomarker with overall survival in the Gd-EOB-DTPA cohort.

Overall survival (n = 72)							
	Univariate analysis				Multivariable analysis (C-index = 0.69)		
Factor	c-index	HR	95% CI	P Value	HR	95% CI	P Value
Age (years)							
≥ 60	0.54	0.94	0.40, 2.19	.89	1.24	0.52, 3.00	.63
< 60	–	–	–	–	–	–	–
Sex							
Male	0.55	1.39	0.60, 3.22	.44	–	–	–
Female	–	–	–	–	–	–	–
Number of tumors							
>1	0.57	1.72	0.67, 4.40	.26	1.52	0.57, 4.03	.40
=1	–	–	–	–	–	–	–
Radiomic risk score							
High^†^	0.66	3.54	1.53, 8.21	.003*	3.23	1.38, 7.65	.008*
Low^†^	–	–	–	–	–	–	–

c-index, concordance index; HR, hazard ratio; CI, confidence interval.

*with p value that indicates statistical significance.

-denotes reference groups.

^†^high and low risk groups were determined using the Maximally Selected Rank Statistic.

**Table 4:** Cox regression model results for the association of radiomics biomarker with overall survival in the Gd-DTPA cohort.

Overall survival (n = 28)							
	Univariate analysis				Multivariable analysis (C-index = 0.74)		
Factor	c-index	HR	95% CI	P Value	HR	95% CI	P Value
Age (years)							
≥60	0.68	0.27	0.09, 0.79	.02*	0.10	0.02, 0.43	.002*
<60	–	–	–	–	–	–	–
Sex							
Male	0.54	0.40	0.09, 1.77	.23	–	–	–
Female	–	–	–	–	–	–	–
Number of tumors							
>1	0.57	1.57	0.57, 4.37	.38	0.77	0.24, 2.45	.66
=1	–	–	–	–	–	–	–
Radiomic risk score							
High^†^	0.64	3.16	1.08, 9.19	.04*	7.78	1.79, 33.73	.006*
Low^†^	–	–	–	–	–	–	–

c-index, concordance index; HR, hazard ratio; CI, confidence interval.

* with p value that indicates statistical significance.

- denotes reference groups.

^†^ high and low risk groups are determined using the Maximally Selected Rank Statistic.

To assess whether the generalizability of our radiomic signature can be attributed to our radiomic feature selection approach, we also used LASSO, a popular technique for feature selection in radiomics, to select features associated with OS in the Gd-DO3A-butrol discovery cohort. Grey level non-uniformity was the only feature selected (texture feature; HR=1.45, p=.01, log-rank test). We also evaluated two features (first-order minimum and small area emphasis, Shur et al.) that have been reported to associate with CLRM prognosis in Gd-EOB-DTPA MRI scans in a recent study (17). Analogous to our radiomic signature, the LASSO feature and Shur et al. selected features were used to build two signatures using logistic regression based on the Gd-DO3A-butrol discovery cohort. Maximally Selected Rank Statistics was applied to find the dichotomization cutoff for other cohorts, resulting in a threshold of 0.30 for Gd-EOBDTPA and 0.26 for Gd-DTPA for our signature. As a result, the high-risk patient group defined by our model is 0.48*MinorAxis +0.15*DependenceVariance−0.86>0.5, 0.3, 0.26 for Gd-DO3A-butrol, Gd-EOB-DTPA and Gd-DTPA, respectively. Thresholds for the other two signatures for the validation cohorts were obtained in the same way. When evaluated for generalizability in the Gd-EOB-DTPA and Gd-DTPA datasets, the model based on features from Shur et al. was only prognostic in the Gd-EOB-DTPA cohort, which is the contrast agent the features were originally proposed in (C-index 0.58; HR=3.45; 95%CI=1.13-10.53; p=.03), and neither model was predictive in the Gd-DTPA cohort. Only our radiomic signature was significantly prognostic in both validation cohorts (**Table 5**).

**Table 5:** Comparison of generalizability of biomarkers when applied to different contrast agents.

Overall Survival									
		Trained on Gd-DO3A-butrol (n=81) Validated on Gd-EOB-DTPA (n=72)				Trained on Gd-DO3A-butrol (n=81) Validated on Gd-DTPA (n=28)			
Factor		C-index	HR	95% CI	P Value	C-index	HR	95% CI	P Value
Grey level non-uniformity^†^									
High risk^ϕ^	0.55		1.55	0.55, 4.34	.41	0.63	2.82	0.99, 8.08	.05
First-order minimum + small area emphasis^‡^									
High risk^ϕ^	0.58		3.45	1.13, 10.53	.03*	0.59	2.30	0.82, 6.40	.11
Minor axis length + dependence variance (our radiomic signature)									
High risk^ϕ^	0.66		3.54	1.53, 8.21	.003*	0.64	3.16	1.08, 9.19	.001*

C-index, concordance index; HR, hazard ratio.

* with p value that indicates statistical significance.

^†^ feature selected using least absolute shrinkage and selection operator (LASSO).

^‡^ features reported in Shur et al’s radiomic analysis for predicting CRLM prognosis in Gd-EOB-DTPA-enhanced MRI.

^ϕ^ high risk groups are determined using the Maximally Selected Rank Statistic.

## 4 Discussion

A few studies have built predictors of long-term prognosis for patients with resected colorectal cancer liver metastases (CRLM) from MRI findings and radiomic features (9, 10, 17, 19). Findings and features were usually selected through correlations with survival. However, the reproducibility and generalizability of these markers have been limited, likely due to the inter-institution variability in MRI protocols. In our study, we excluded radiomic features that describe tumor intensity as they are likely heavily influenced by contrast agent choice. We also selected a feature in each of the remaining radiomic feature categories: shape and texture, and ensured that the two selected features were not correlated in the discovery cohort. Our radiomic signature model based on minor axis length (shape feature) and dependence variance (texture feature) not only validated in the validation cohort (Gd-EO3A-butrol, n=40, HR=6.29, p=.007) and an independent cohort using a different contrast agent from the same institute (Gd-EOB-DTPA, n=72, HR=3.54, p =.003), but also a cohort using a third contrast agent from an independent center (Gd-DTPA, n=28, HR=3.16, p=.04).

Although CRLMs appear qualitatively very different on delayed phase with extracellular contrast agents (Gd-DO3A-butrol and Gd-DTPA) as compared to hepatobiliary-specific contrast agents (Gd-EOB-DTPA), this is largely due to differences in background hepatic uptake. Because the tumors themselves do not contain hepatocytes and therefore do not actively take up contrast, their enhancement characteristics are likely very similar in both types of contrast agents. This could explain why radiomics models that focus on segmented tumors might be generalizable across different contrast agents. While intensity features vary considerably due to changes in the absolute intensity of voxels, texture features, which could reflect intra-tumor heterogeneity (20), remain relatively stable across different contrast agents. The ability of our radiomics signature to be robust across different contrast agents and institutions suggests that it is possible to build a predictive biomarker for DPCE MRI of CRLM patients based on only shape and texture features, and its applicability may be relatively generalizable. Further studies are required to validate this in larger datasets.

In this study we looked at MRIs enhanced with three different contrast agents and acquired using different imaging parameters. Gd-EOB-DTPA is increasingly the contrast agent of choice for staging of CRLM, although Gd-DO3A-butrol is still used in many instances for diagnosing focal liver lesions and remains used for staging in some institutions. Gd-DTPA has subsequently been discontinued due to increased risk of nephrogenic systemic fibrosis. However, the purpose of our study is not to develop a signature for these three contrast agents but to demonstrate that radiomic biomarkers can be designed to be more generic to avoid overfitting to specific imaging protocols.

There are several limitations in our study. First, this study is a preliminary, retrospective study that investigated the overall survival of patients with resected CRLM, which is a highly selective cohort of CRLM patients. A future work with a radiomic biomarker that is also predictive for unresectable CRLM patients would be beneficial to a broader patient population. Second, the external Gd-DTPA dataset is relatively small. Prospective studies with large enrolments are required to validate the radiomic signature proposed and determine its clinical value. Third, there exist variations in the exact amount and types of preoperative chemotherapy in this retrospective study, which could affect the results. Also, tumor segmentation was performed by a single reader in each cohort and further studies investigating segmentation inter-rater reliability is required.

In conclusion, in patients with resectable CRLM, a logistic regression model based on radiomic features discovered and trained on a Gd-DO3A-butrol discovery cohort was shown to be prognostic in three multi-contrast, multi-center cohorts. While most existing MRI findings focus on a specific contrast agent, our study shows the potential of MRI features to be generalizable across main-stream contrast agents at delayed phase.

## Data availability statement

The raw data supporting the conclusions of this article will be made available by the authors, without undue reservation.

## Ethics statement

The studies involving human participants were reviewed and approved by The Research Ethics Board of Sunnybrook Health Sciences Center. Written informed consent for participation was not required for this study in accordance with the national legislation and the institutional requirements.

## Author contributions

HC, CP, LM and AM conceptualized and designed the study. JC, HC, PK, NC, AL, CP and LM acquired the data. JC and HC analyzed and interpreted the data. JC and HC drafted the manuscript. All authors contributed to the article and approved the submitted version.

## Footnotes

1
https://pyradiomics.readthedocs.io/en/latest/features.html#module-radiomics.shape

2
https://pyradiomics.readthedocs.io/en/latest/features.html#module-radiomics.gldm
