# Supplementary material for: A radiomic biomarker for prognosis of resected colorectal cancer liver metastases generalizes across MRI contrast agents
Source: Front Oncol. 2023 Feb 2;13:898854. doi: 10.3389/fonc.2023.898854 (PMC9932499; doi:10.3389/fonc.2023.898854)
Supplement: Supplementary file 1 [file DataSheet_1.pdf]

Table S1: Parameters and for radiomic feature extractions. A list of radiomic feature class is also attached.

**Parameters**	DichotomizationBinWidth:5, Interpolator: 'sitkBSpline', ResamplePixelSpacing: [1.5, 1.5, 1.5],
**FeatureClass**	**Shape**: Elongation, Flatness, LeastAxisLength, MajorAxisLength, Maximum2DDiameterColumn, Maximum2DDiameterRow, Maximum2DDiameterSlice, Maximum3DDiameter, MeshVolume, MinorAxisLength, Sphericity, SurfaceArea, SurfaceVolumeRatio, VoxelVolume<br>**Firstorder**: 10Percentile, 90Percentile, Energy, Entropy, InterquartileRange, Kurtosis, Maximum, Mean, MeanAbsoluteDeviation, Median, Minimum, Range, RobustMeanAbsoluteDeviation, RootMeanSquared, Skewness, TotalEnergy, Uniformity, Variance<br>**glcm**: 'Autocorrelation', 'JointAverage', 'ClusterProminence', 'ClusterShade', 'ClusterTendency', 'Contrast', 'Correlation',<br>'DifferenceAverage', 'DifferenceEntropy', 'DifferenceVariance', 'JointEnergy', 'JointEntropy', 'Imc1', 'Imc2', 'Idm', 'Idmn', 'Id', 'Idn',<br>'InverseVariance', 'MaximumProbability', 'SumEntropy', 'SumSquares'<br>**glrlm**: all; **glszm**: all; **gldm**: all;
